# A Micro-Damage Detection Method of Litchi Fruit Using Hyperspectral Imaging Technology

**DOI:** 10.3390/s18030700

**Published:** 2018-02-26

**Authors:** Juntao Xiong, Rui Lin, Rongbin Bu, Zhen Liu, Zhengang Yang, Lianyi Yu

**Affiliations:** College of Mathematics and Informatics, South China Agricultural University, Guangzhou 510642, China; limyui@stu.scau.edu.cn (R.L); bobby@stu.scau.edu.cn (R.B); liuz@stu.scau.edu.cn (Z.L); yzg@ scau.edu.cn (Z.Y); scauyly@163.com (L.Y.)

**Keywords:** hyperspectral imaging, litchi quality, micro-damages detection, image recognition

## Abstract

The non-destructive testing of litchi fruit is of great significance to the fresh-keeping, storage and transportation of harvested litchis. To achieve quick and accurate micro-damage detection, a non-destructive grading test method for litchi fruits was studied using 400–1000 nm hyperspectral imaging technology. The Huaizhi litchi was chosen in this study, and the hyperspectral data average for the region of interest (ROI) of litchi fruit was extracted for spectral data analysis. Then the hyperspectral data samples of fresh and micro-damaged litchi fruits were selected, and a partial least squares discriminant analysis (PLS-DA) was used to establish a prediction model for the realization of qualitative analysis for litchis with different qualities. For the external validation set, the mean per-type recall and precision were 94.10% and 93.95%, respectively. Principal component analysis (PCA) was used to determine the sensitive wavelength for recognition of litchi quality characteristics, with the results of wavelengths corresponding to the local extremum for the weight coefficient of PC3, i.e., 694, 725 and 798 nm. Then the single-band images corresponding to each sensitive wavelength were analyzed. Finally, the 7-dimension features of the PC3 image were extracted using the Gray Level Co-occurrence Matrix (GLCM). Through image processing, least squares support vector machine (LS-SVM) modeling was conducted to classify the different qualities of litchis. The model was validated using the experiment data, and the average accuracy of the validation set was 93.75%, while the external validation set was 95%. The results indicate the feasibility of using hyperspectral imaging technology in litchi postpartum non-destructive detection and classification.

## 1. Introduction

Litchi (litchi chinensis) is a characteristic fruit in southern China, as is famous as a delicacy worldwide. It is prone to deterioration and is not suitable for long-term storage. The litchi pericarp is easily damaged by mechanical collision during picking, transportation and other manual operations. Damaged pericarp can hasten fruit rot, resulting in worse taste [1,2,3]. According to measurements, 100 g of litchi contains 16.53 g of carbohydrates, 15.23 g of sugar, 0.83 g of protein and 71.5 mg of vitamin C. The scent of litchi is maintained by phenolics. With an increase in storage time, the contents of total phenolics and free phenolics decrease continuously, resulting in worsening taste and flavor of the litchi [4,5]. Using information technology to measure and determine the freshness of litchi fruit is of great significance to the post-harvest quality grading and preservation of litchi.

In recent years, with the development of new information technology, hyperspectral imaging technology and chemometrics methods are increasingly applied to food, medicine, and agricultural environments and production areas [6,7,8,9]. Hyperspectral imaging technology has the advantages of non-destructive detection, the ability to analyze more qualities simultaneously, and the ability to analyze the interior and exterior qualities of cereal, such as ergot bodies or protein content [10,11].

Hyperspectral imaging technology has been used to detect damage in fruits. In order to detect early bruising in apples, Baranowski et al. [12] used a system tha incorporated the hyperspectral imaging of reflected radiation in visible and near-infrared (VNIR) and short-wavelength infrared (SWIR) ranges and infrared thermal imaging of emitted radiation in the mid-wavelength infrared (MWIR) range to detect bruises created one hour before their experiment. They confirmed that broad spectrum range (400–5000 nm) surface imaging of fruit can improve the detection of early bruises with varying depths. Cen et al. [13] acquired the hyperspectral reflectance (400–700 nm) and transmittance (700–1000 nm) images to identify three-class (normal, slightly defective, and severely defective) classifications of pickling cucumbers. ElMasry et al. [14] investigated hyperspectral imaging (400–1000 nm) and artificial neural network (ANN) techniques to detect chilling injury in Red Delicious apples. Niphadkar et al. [15] selected a two-band ratio method with a simple threshold-based classifier (ratio of reflectance at wavelengths 834 nm and 729 nm) to estimate the lesion size of citrus. In order to estimate internal properties or detect invisible damage, Rivera et al. [16] acquired the images of damaged and intact areas of mangos in the range 650–1100 nm using a hyperspectral computer vision system. Folch-Fortuny et al. [17] developed an N-way partial least squares regression discriminant analysis (NPLS-DA) methodology to detect symptoms of disease caused by *Penicillium digitatum* in citrus fruits (green mould) using VNIR hyperspectral images.

Hyperspectral imaging technology has been applied to the study of litchis as well. In order to investigate the ability of the hyperspectral imaging technique and multivariate classification for the differentiation of litchis’varieties, Liu et al. [18] used linear (soft independent modeling of class analogy (SIMCA) and partial least square discriminant analysis (PLS-DA)) and nonlinear (back propagation neural network (BPNN) and support vector machine (SVM)) multivariate classification methods to develop discrimination models. Yang [19] used hyperspectral imaging to explore the relationship between browning levels of litchis and moisture contents (MC) of pericarp, and developed calibration models for determining browning degree of litchis based on the MC prediction of pericarp. For predicting the polyphenol oxidase (PPO) activity in litchi pericarp, Yang [20] used a hyperspectral reflectance imaging system operating in the range of 400–1000nm to capture the litchi images, and combined it with a fuzzy neural network (FNN). This work showed that the hyperspectral imaging technique could be used to predict the internal and external quality attributes of fruits. The existing research utilizing hyperspectral image technology is able to predict the quality and conduct damage detection for fruits, which is of great significance for practical application in agricultural production.

To date, little research related to the damage feature identification of litchi using hyperspectral imaging has been done. The object of this study was to investigate the potential of hyperspectral imaging for micro-damage identification of fresh litchi damage features. The specific objects of the study were (1) employing a lab hyperspectral imaging system with the proper wavelength to acquire the hyperspectral images of litchis in different qualities, (2) establishing a PLS-DA model for the qualitative analysis and detection of litchi quality using hyperspectral imaging, (3) extracting the characteristic wavebands for the classification of damaged litchi using principal component analysis (PCA) and computing the parameters of Gray Level Co-occurrence Matrix (GLCM) based on image processing for qualitative identification of litchis’ damage condition, (4) establishing qualitative discrimination models using least squares support vector machines (LS-SVM) for the classification of litchi quality.

## 2. Materials and Methods

### 2.1. Test Materials and Equipment

**Figure 1:**
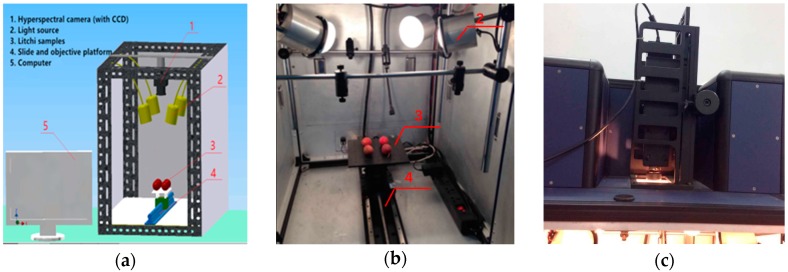
Hyperspectral image acquisition system. (**a**) Sketch map, (**b**) Physical picture, (**c**) Hyperspectral camera with CCD.

The Huaizhi litchi, selected as the test object in this study, is planted in Guangdong province of China with a maturation period between the end of June and mid-July. The experiment was implemented by a litchi hyperspectral image acquisition system with a hyperspectral imager (Hyper SIS VNIR-QE, Zolix Instruments Co., Ltd., Beijing, China). The imaging system, with factory settings, consists of a light source with four halogen lamps, a hyperspectral camera with CCD, a camera obscura, a mobile sample platform and a computer. The image acquisition system has a spectral wavelength measurement range of 300–1000 nm, a resolution of 2.8 nm, exposure time of 15 ms, and a conveyor belt transmission speed of 5 mm/s. Its structure is shown as Figure 1. The hyperspectral image data acquisition software is Spectra SENS (Gilden Photonics Ltd., Glasgow, UK).

### 2.2. Test Method

Spectra SENS software was used for hyperspectral image acquisition, and the software package with ENVI V4.7 (Research System, Inc., Boulder, CO, USA), Matlab 2012, Microsoft Excel 2010 and Unscrambler X10.3 were used for the test data analysis. Before the hyperspectral image acquisition, the exposure time of the hyperspectral cameras was predetermined to ensure the clarity of the image, and the speed of the transmission device was determined to avoid distortion of the image size and spatial resolution. In order to acquire clear and undistorted hyperspectral images, several parameters of the device need to be adjusted before acquiring the images [21]. White reflectance and dark current images were acquired to correct the obtained sample images. White reflectance images were acquired using a white board with approximately 99% reflectance, while dark reflectance images were obtained by covering the lens. To acquire the relative reflectance hyperspectral images of litchi samples, original hyperspectral image data were conducted using following Equation (1).

(1) $$I=\frac{I_{0}-D}{W-D},$$

where *I* is the relative reflectance hyperspectral image, *I*_0_ is the original image data, *W* is the white reflectance image data, and *D* is the dark current image data.

### 2.3. Test Procedure

The purpose of this experiment is mainly to collect hyperspectral image information of fresh and damaged litchi. The adopted litchi fruits are freshly picked from the orchard using a fixed storage device for transportation. Litchi is prone to damage, such as transportation squeeze damage, natural cracks, and mildew. This study focuses on the conditions of litchi epidermis micro-damage that have an inconspicuous appearance; thus, the conditions of cracking and mildew, which have obvious appearances, are not included.

The fruit of Huaizhi litchi, with a longitudinal diameter (distance from pedicel to fruit top) of 29.60–32.56 mm and two mutually perpendicular horizontal diameters of 29.20–33.46 mm and 29.24–34.00 mm, was selected for the mechanical damage simulation test. Litchis picked at the same time were selected to maintain fruit status. A precision, micro controlled, electronic universal testing machine WD-20KE (YDYQ Precision Instruments Co., Ltd., Guangzhou, China) was used to automatically acquire data regarding force and displacement and had a precision of ±0.5%, a resolution ratio of ±1/120,000, and a sampling frequency of 5 Hz. The rigid plate compression method was used for the fruit compression test, with the top board vertically pushing down and a fixed lower plate. The loading rate is 45 mm/min. With the increase of load force, the litchi pericarp ruptures, and the corresponding loading force is recorded as the litchi rupture strength and the time is recorded as the rupture point. The stress and deformation of the rupture point were then analyzed, and the compression ratio of the rupture point was calculated according to the diameter of the litchi, with the result being used as a reference index for the litchi mechanical damage test. The test process is as follows.

**Figure 2:**
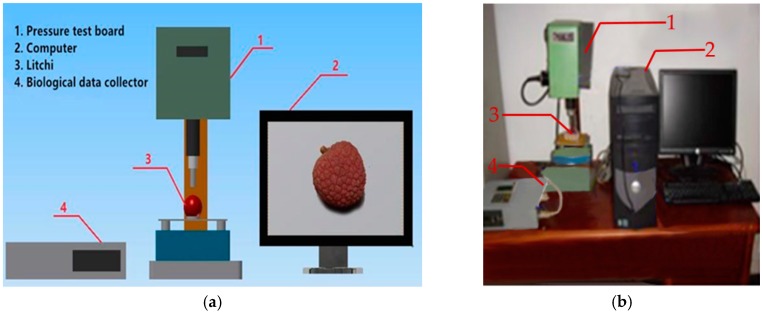
The test platform for data acquisition of litchi damage. (**a**) Sketch map, (**b**) Physical picture.

Step 1: Litchi pericarp quality screening. In the litchi maturation period of July, 300 fresh litchi fruits were picked randomly from the same tree in the litchi garden of Zengcheng, Guangzhou. The fruits were stored in a refrigerator set on 3 °C and transported to the laboratory. Then the fruits were exposed to normal room temperature for 45 min. The microscope was used to remove fruits with cracks or damages caused by collision or squeeze. Litchi fruits with intact pericarp were selected as samples. Some litchis were used to simulate mechanical damage by the pressure test bench shown as Figure 2. Damaged litchis confirmed by the microscope were selected as micro-damage samples.

Step 2: Perform hyperspectral image acquisition of intact litchi and damaged litchi. The hyperspectral data of intact litchis were collected. Once an image had been collected, the litchi was turned clockwise 90° along the equator for another image acquisition. Namely, each litchi was measured four times. The data of damaged litchi were collected in the same way. After data collection, both kinds of litchis were left at room temperature.

Step 3: Perform hyperspectral image acquisition of damaged litchis after a certain period of time. The damaged litchis were left at room temperature. The data collection was performed every 30 min using the procedure in Step 2. This step lasted for 4 h. Then the images of the intact litchi, the newly damaged litchis, the damaged litchis after 2 h and the damaged litchis after 4 h were chosen for analysis.

**Figure 3:**
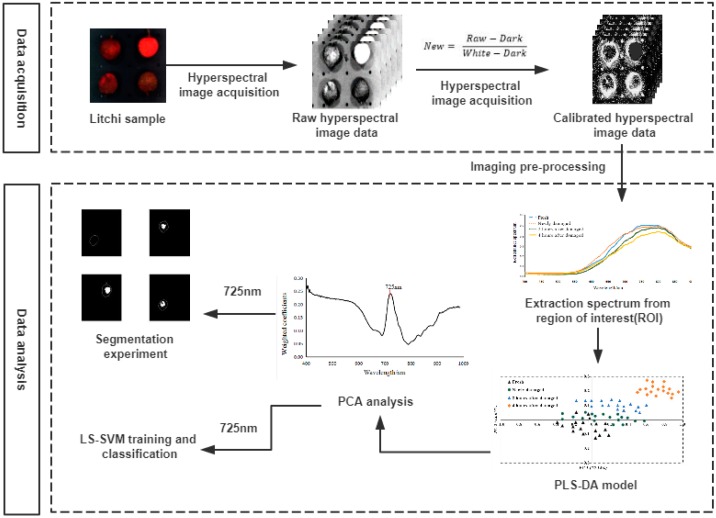
Diagram of the main procedure for analyzing hyperspectral imaging data.

Figure 3 illustrates the critical procedure for analyzing the hyperspectral imaging data. Spectral information obtained from the hyperspectral image acquisition system was used for disclosing the distinction between fresh ripe litchi and micro-damage litchi. Original spectra were pre-processed to extract the region of interest of a target in a litchi. Image acquisition, calibration, spectral data extraction, and data analysis were performed by MATLAB software (version 2012b, Mathworks, Natick, MA, USA).

## 3. Results and Discussion

### 3.1. Spectral Curve Analysis of Litchis of Different Quality

**Figure 4:**
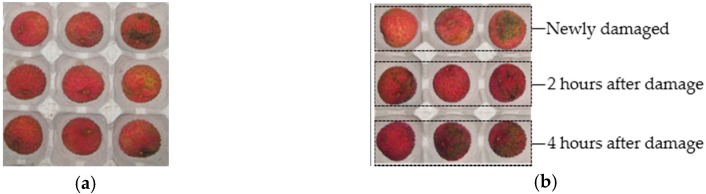
Litchi images of different qualities. (**a**) Fresh mature litchis, (**b**) Micro-damage litchis of different qualities.

As we know from the biological characteristics of litchi, the epidermis color of immature, mature and damaged litchis will change gradually from red to brown after picking. Damaged litchi turns brown the fastest, mature litchi the second fastest, and immature litchi the slowest. This study aimed to research the hyperspectral imaging detection technology for mature litchi in postpartum damaged state. Two different qualities of litchi fruit are shown as Figure 4.

The samples of litchis of four different qualities—fresh, newly damaged, 2 h after damage and 4 h after damage—were selected, with 30 fruits for each quality (the newly damaged, 2 h after damage and 4 h after damage were the same 30 litchis at different times). Then, hyperspectral data were acquired, and the average was calculated for the 30 litchis of each quality. The hyperspectral images were pre-processed using the image acquisition system to obtain the region of interest (ROI, a 50 × 50-pixel window containing the area of the litchi pericarp damage) of a target in a litchi hyperspectral image, and a micro-damage rupture site containing litchi skin in the ROI was identified. This paper set the window size to 50 × 50 pixels, and the average hyperspectral reflectance value within the wavelength range of 400–1000 nm in the window was calculated. The spectral data were processed to reduce instrument and ambient noise, and the influence of the uneven samples. Here, we mainly carried out smoothing, de-noising, baseline correction and de-trend processing on the spectral data, as well as selective SG (Savitzky-Golay) smoothing [22].

**Figure 5:**
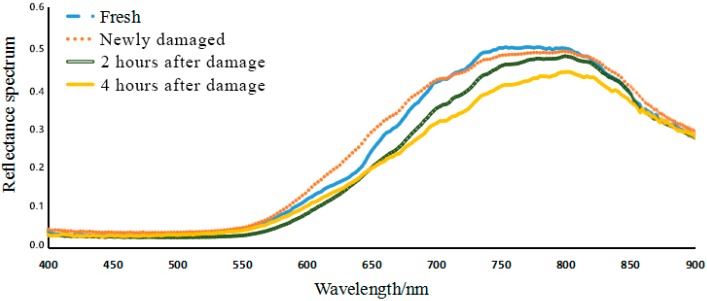
Hyper-spectral reflectance curves of different quality litchis.

The hyperspectral data curves for the four qualities of litchi are shown in Figure 5. The hyperspectral image data have obvious noise when the wavelength is below 450 nm or above 850 nm, and the hyperspectral curves have obvious peaks and troughs with curve smoothing within the wavelength range of 450–850 nm. In addition, the hyperspectral reflectance spectrum curves of different quality litchis contain differences that showing the spectral reflectance data of different quality litchis being different under hyperspectral excitation light.

### 3.2. Qualitative Analysis of Litchi Fruit Quality

PLS-DA is a supervised pattern recognition method not only for orthogonally analyzing the measurement matrix, but also for orthogonally decomposing the response matrix at the same time. It is a method of regression based on characteristic variables. PLS-DA for the spectrum of litchi fruit was performed by calibrating samples, and the spectral feature PLS-DA model was established, using the internal leave-one-out method for full cross-validation.

120 samples from three independent experiments (i.e., 40 samples per batch) were used to develop the model, and 80 samples from the fourth and the fifth experiment were employed for the purpose of external validation. It should be noted that 200 samples from different batches were analyzed in total. From this point on, litchi samples from the previously mentioned independent experiments will be referred to as samples from batches 1, 2, 3, 4 and 5. As the method being supervised, the data were partitioned in two sets, a training set used for model calibration and a test set used for validation. A 60/40% stratified partition was applied to the first three batches, meaning 60% of the dataset was chosen in a random way for calibration (72 samples out of 120), as long as all types and batches were included and equally represented. The fourth and the fifth batches were also reserved for independent model validation.

Litchis of four qualities—fresh, newly damaged, 2 h after damage and 4 h after damage—were selected for the forecast model calibration, with corresponding sample values of 0, 1, 2, and 3.

**Figure 6:**
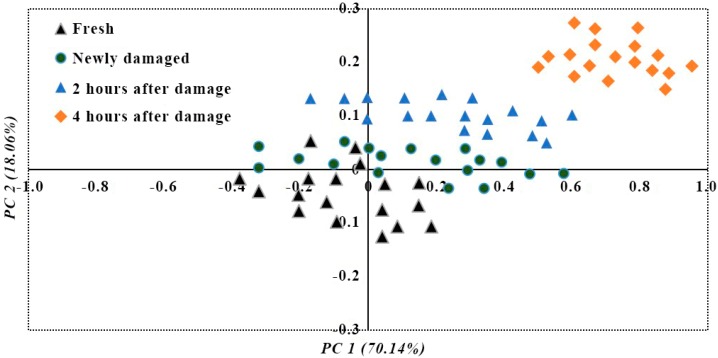
Qualitative analysis of different qualities of litchis by PLS-DA model.

The hyperspectral data from 72 litchi images were used for PLS-DA modeling analysis, and the latent variable score plot of the four kinds of images are shown in Figure 6. The forecasting model obviously distinguished among the four qualities of litchis. There was a clear distribution and area of distinction between 4 h after damage and 2 h after damage and the other qualities of litchi, with fresh litchi and newly damaged litchi in the normal distribution area, adjacent to one another, and crossing to a certain extent.

The performance of the PLS-DA model was measured in terms of recall (sensitivity) and precision [23] to predict the quality of the litchis. The analysis of data acquired for each type of litchi is shown as Table 1, where the results of recall and precision for the different samples are presented. The mean per-type recall and precision were 92.19% and 91.74%, respectively. In the case of external validation, PLS-DA performed well for correctly classifying all images of the four types of litchis. In this case, the mean per-type recall and precision were 94.10% and 93.95%, respectively.

### 3.3. Characteristic Band Selection of Litchi Hyperspectral Images

**Figure 7:**
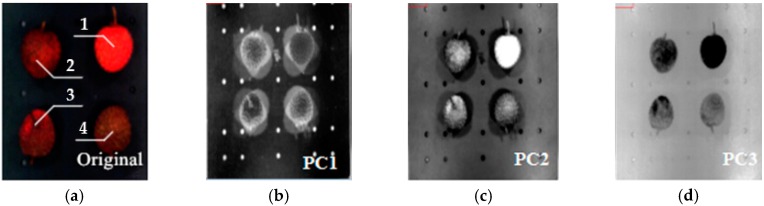

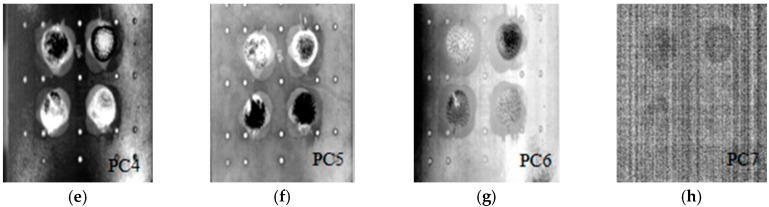
Litchi fruit images by principal component analysis. (**a**) Original image 1. Fresh, 2. Newly damaged, 3. 4 h after damage, 4. 2 h after damage, (**b**–**h**) Principle components 1 to 7.

In this study, the image PCA analysis method is used to select the characteristic band. Image PCA analysis is a method for linear transformation of multi-band data from the spectral data to a new coordinate system, maximizing the data disparity to determine the characteristic band. The multi-band spectral data were analyzed by PCA to obtain corresponding covariance matrix of the spectral data, and the weight coefficients of each principal component were calculated. Figure 7 shows the PC1–PC7 images obtained from the principal component analysis of the spectral samples of four qualities of litchis in the range of 400–1000 nm.

The contribution rates of seven PCs are listed in Table 2. The cumulative contribution rate of seven PCs reaches 99.89%, which means that these 7 PCs can explain 99.89% of the information content of the raw spectral data.

**Figure 8:**
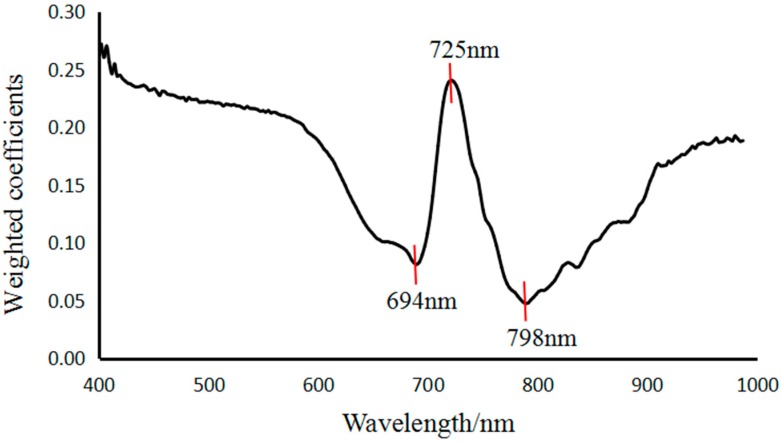
Weighted coefficient of PC3 image.

As shown in Figure 7, there is more noise in PC7, and PC1–PC6 are better able to preserve the integrity of information regarding the surface of litchi fruits. Using digital image processing knowledge to analyze each PC image histogram, we found that in the PC3 histogram of different quality litchis, there are obvious peaks and valleys, making it suitable for image recognition of litchi quality. Overall, PC3 preserved relatively complete information regarding different quality litchis. Therefore, if we choose the weight coefficient of the PC3 image to select the characteristic band, shown in Figure 8, the characteristic bands would be determined as 694, 725 and 798 nm by the PLSR model [24].

**Figure 9:**
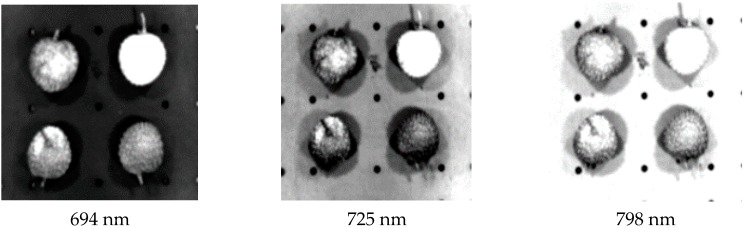
Single-band greyscale image based on sensitive bands of PC3.

We analyzed the single-band images of the characteristic bands corresponding to the local extremum of PC3 weight coefficients, shown as Figure 9. Based on the analysis of the grey scale histogram of the images of the 3 characteristic bands, we found the 798 nm images to contain more noise, and both the 694 nm and 725 nm images to have a clear distinction between fresh and damaged litchis. From the single-band images of 694 and 725 nm, we know the characteristics of different degrees of damaged litchis are even more obvious in the single-band image of 725 nm. In summary, the single-band image of 725 nm is the best image for identification of different quality litchis.

### 3.4. Test Analysis of LS-SVM Model Based on Image Processing

The hyperspectral data was used to establish a prediction model that can be used for the identification of litchi quality, and hyperspectral image processing can provide more accurate information regarding the quality of litchis. Hyperspectral images of different quality litchis were collected, and the greyscale images of litchis under a 725 nm wave band were selected to identify the quality of litchi fruits. 

**Figure 10:**
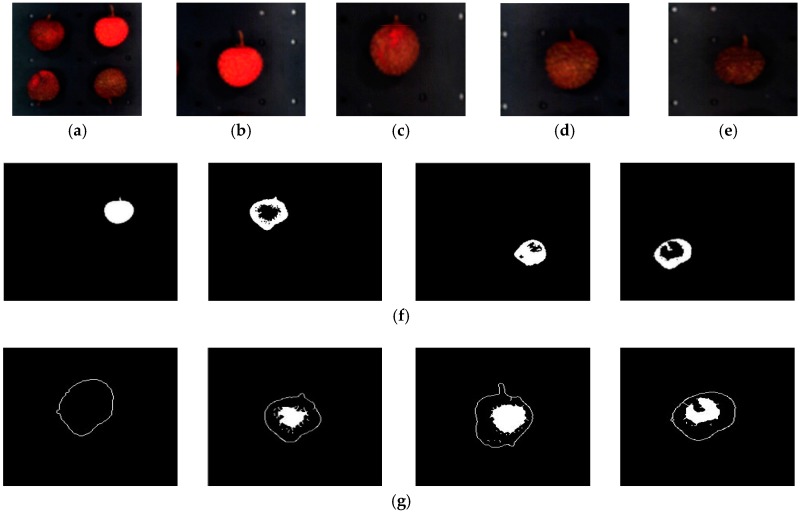
Image processing results of different qualities of litchis. (**a**) Hyperspectral image, (**b**) Fresh, (**c**) Newly damaged, (**d**) 2 h after damage, (**e**) 4 h after damage, (**f**) Results of Otsu adaptive threshold segmentation (left to right), Fresh, newly damaged, 2 h after damage, 4 h after damage, (**g**) Results of edge detection and fuzzy clustering (left to right), Fresh, Newly damaged, 2 h after damage, 4 h after damage.

The positioning of litchi fruit and the removal of background can be helpful to the acquisition of a single fruit, whose characteristics can be used for further training and prediction. The PC3 image of hyperspectral data from PCA analysis was selected for the equalization preprocessing of the grayscale image histogram to remove noise. Then, Otsu adaptive threshold segmentation [25] and edge detection with Robert operator [26] were used to locate the single litchi, as shown in Figure 10. Finally, the damaged parts were obtained by fuzzy clustering [27]. The image processing results for different qualities of litchi are shown in Figure 10. 

LS-SVM [28], a typical and effective prediction model, was used in further analysis of litchi qualities. As litchi pericarp is damaged, the texture and color change. Therefore, GLCM [29] was used to extract the 7-dimensional texture features, including contrast, correlation, energy, homogeneity, variance, mean value (the mean value can express the color characteristics of the image) and entropy. These features were then used for further analysis and the modeling.

The parameters of GLCM were computed based on the image processing results of litchis in different qualities. Since the 7-dimensional texture features are difficult to visualize with figures, this paper analyzes the features of the training samples with a table. As shown in Table 3, each unit represents the average value of a certain type of sample in a certain dimension. It can be seen that these 7-dimensional texture features have a certain degree of discrimination for sample classification.

72 of the 120 images (30 images for each quality) were used to train the LS-SVM model. The remaining 48 images were used as a validation set. Another 80 images (20 images for each quality) were used as an external validation set. Results are shown in Table 4. It can be seen that, for the validation set, the highest accuracy reached 100%. The average accuracy for the validation set was 93.75%. For the external validation set, the average accuracy was 95.00%. This is similar to the result of the validation set, proving the efficiency of the LS-SVM model used in this paper.

## 4. Conclusion and Future Work

A non-destructive testing method of litchi fruit quality was realized using hyperspectral imaging technology.

(1) By analyzing hyperspectral data of different quality litchis using the PSL-DA method to establish prediction model, qualitative analysis of litchi fruit quality based on hyperspectral data was realized. For the validation set, mean per-type recall and precision were 92.19% and 91.74%, respectively. For the external validation set, the mean per-type recall and precision were 94.10% and 93.95%, respectively. This indicates that hyperspectral imaging technology is feasible for realizing non-destructive testing of litchi fruit.
(2) By determining the optimum band for image recognition of litchi quality with principal component analysis, the 725 nm band image was selected, which corresponds to the weight coefficient of PC3. The LS-SVM was established based on the 7-dimensional texture features of GLCM. The average accuracy of the validation set was 93.75%, while the external validation set was 95%.

In conclusion, this study provides a theoretical basis and a technical reference for the quality classification of the same type of fruits and vegetables based on the technology of litchi quality detection using hyperspectral imaging. In order to achieve the on-line detection of litchi fruit quality, future work will focus on exploration and evaluation of improved techniques for application.

## Figures and Tables

**Table 1:** PLS-DA performance for validation set and external validation set with 4 types of litchis.

Sample	Validation Set (%)		External Validation Set (%)	
	Recall	Precision	Recall	Precision
Fresh	94.18	100.00	100.00	95.27
Newly damage	90.23	85.71	88.11	91.14
2 h after damage	91.21	90.18	94.28	93.24
4 h after damage	93.15	91.07	94.00	96.14
Mean per-type	92.19	91.74	94.10	93.95

**Table 2:** Cumulative contribution rate of seven principal components.

No. of PC	PC 1	PC 2	PC 3	PC 4	PC 5	PC 6	PC 7
Percent (%)	70.14	88.20	96.61	98.17	99.05	99.61	99.89

**Table 3:** Averaged textural features extracted by GLCM.

Type of Detection	Contrast	Correlation	Energy	Homogeneity	Variance	Mean	Entropy
Fresh	186.301	1.520	0.129	1.787	6.778	0.505	4.212
Newly damaged	200.885	1.925	0.125	1.532	5.264	0.171	5.133
2 h after damaged	178.488	2.418	0.319	2.401	4.027	0.102	2.899
4 h after damaged	264.357	1.900	0.209	2.633	5.078	0.039	4.821

**Table 4:** Discrimination results for different quality litchis with confusion matrix.

Sample	Validation Set					External Validation Set				
	Fresh	Newly Damaged	2 h after Damage	4 h after Damage	Accuracy	Fresh	Newly Damaged	2 h after Damage	4 h after Damage	Accuracy
Fresh	12	0	0	0	100.00	20	0	0	0	100.00
Newly damaged	1	10	1	0	83.33	2	18	0	0	90.00
2 h after damage	0	1	11	0	91.67	0	2	18	0	90.00
4 h after damage	0	0	0	12	100.00	0	0	0	20	100.00

Note: the columns refer to the actual discrimination of samples, the rows refer to the discrimination by model.
